# Prolonged dexmedetomidine infusion and drug withdrawal in critically ill children

**DOI:** 10.1186/cc14564

**Published:** 2015-03-16

**Authors:** A Haenecour, A Goodwin, W Seto, C Urbain, P Laussen, C Balit

**Affiliations:** 1 The Hospital for Sick Children, Toronto, ON, Canada

## Introduction

We investigated the incidence, symptoms and risk factors for withdrawal associated with prolonged dexmedetomidine use. Dexmedetomidine is an α_2_-adrenergic receptor agonist, with anxiolytic, analgesic and sedative properties. Intended for short-term use, there is increasing literature describing prolonged use for sedation. However, this raises the potential of withdrawal syndrome and there is no recommendation for the discontinuation of dexmedetomidine. Other goals included determining the hemodynamic effects of discontinuation of dexmedetomidine and role of clonidine in patients with prolonged dexmedetomidine use.

## Methods

A retrospective review of patients admitted to the critical care unit who had exposure to dexmedetomidine for longer than 48 hours, between 1 January 2014 and 15 July 2014. Data included patient demographics, dexmedetomidine exposure (bolus dose, total cumulative dose, duration), other sedative exposure, withdrawal symptoms measured by WAT-1 score, nursing subjective assessment and treatment given for withdrawal. Each potential withdrawal episode was reviewed by two reviewers. Hemodynamic parameters were analyzed to assess hemodynamic changes associated with discontinuation of dexmedetomidine. Descriptive statistics were used with *t *test and chi-square test. Median and interquartile range (IQR) are reported.

## Results

A total of 53 patients accounted for 69 unique dexmedetomidine treatment courses. Median age at the time of dexmedetomidine infusion was 5 months (range 1 day to 3 years). Dexmedetomidine dose ranged from 0.1 to 2 μg/kg/hour with a median cumulative dose of 87 μg/kg (IQR 53, 156). Median duration of exposure to dexmedetomidine was 124 hours (IQR 76, 178) with a maximum duration of 466 hours. We identified 24 separate episodes of withdrawal (incidence 35%). Most common symptoms were agitation (100%), fever (67%), vomiting/retching (46%), loose stools (29%) and decreased sleep (20%). Statistical analysis showed that factors significantly associated with withdrawal were cumulative dose (*P *= 0.01) and duration of use of dexmedetomidine (*P *= 0.02). Duration of opioids exposure prior to dexmedetomidine wean was also a risk factor for withdrawal (*P *= 0.01). Use of clonidine as a transition from dexmedetomidine did not protect against withdrawal (*P *= 0.59).

## Conclusion

This study showed that withdrawal syndrome is associated with prolonged infusion of dexmedetomidine. Patients with higher cumulative doses and longer duration of exposure were more at risk. Our results suggested that clonidine use is not protective for withdrawal from dexmedetomidine.

